# Supplementary material for: Order of amino acid recruitment into the genetic code resolved by last universal common ancestor’s protein domains
Source: Proc Natl Acad Sci U S A. 2024 Dec 12;121(52):e2410311121. doi: 10.1073/pnas.2410311121 (PMC11670089; doi:10.1073/pnas.2410311121)
Supplement: Supplementary file 1 — Appendix 01 (PDF) [file pnas.2410311121.sapp.pdf]

Supporting Information for
Order of amino acid recruitment into the genetic code resolved by Last Universal Common Ancestor's protein domains

Sawsan Wehbi[1], Andrew Wheeler[1], Benoit Morel[2], Nandini Manepalli[3], Bui Quang Minh[4], Dante S. Lauretta[5], Joanna Masel[6]

[1]Genetics Graduate Interdisciplinary Program, University of Arizona, Tucson, Arizona, 85721, USA

[2]Computational Molecular Evolution Group, Heidelberg Institute for Theoretical Studies, Heidelberg, Germany

[3]Department of Molecular and Cellular Biology, University of Arizona, Tucson, AZ, 85721, USA

[4]School of Computing, Australian National University, Canberra, ACT, Australia

[5]Lunar and Planetary Laboratory, University of Arizona, Tucson, AZ 85721, USA

[6]Department of Ecology and Evolutionary Biology, University of Arizona, Tucson, AZ, 85721, USA

Corresponding author: Joanna Masel

Email: masel@arizona.edu

This PDF file includes:

Figures S1 to S5
Table S1
Legends for Datasets S1 to S2
SI References

Other supporting materials for this manuscript include the following:

Datasets S1 to S2

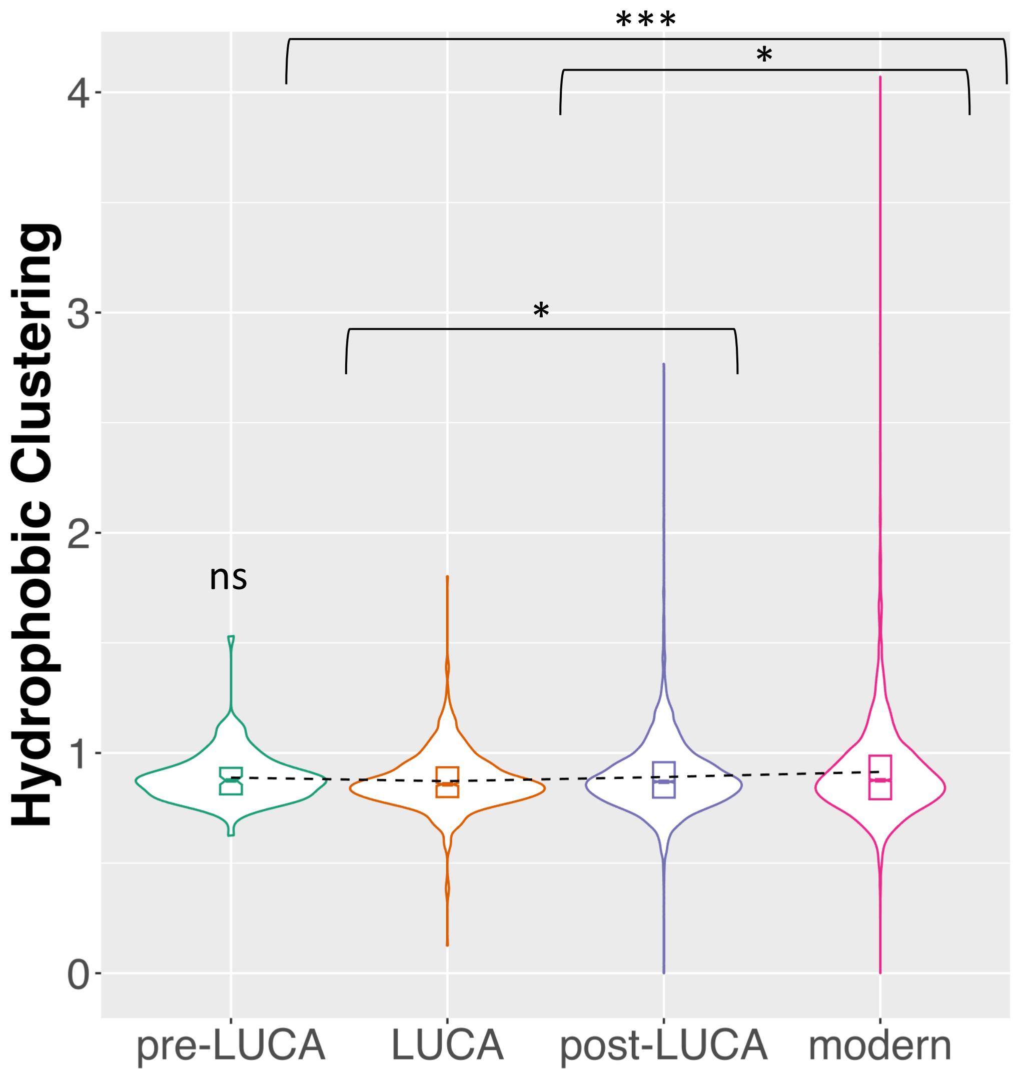

Figure S1. Older protein Pfams have more interspersed hydrophobic amino acids. Independent amino acid placements correspond to a "hydrophobic clustering" score of 1, values less than 1 indicate interspersed hydrophobic amino acids along the primary sequence, values greater than 1 indicate clustered hydrophobic amino acids; see Methods for details. Hydrophobic amino acids are more interspersed (with lower clustering value) in LUCA Pfams than in either post-LUCA Pfams ($p = 0.02$) or modern Pfams ($p = 0.0003$). The latter two also differ from each other ($p = 0.02$). Pairwise Wilcoxon rank sum tests were used to test for statistical significance. Boxplots show the median, upper, and lower quartiles of the data. Violin plots were generated using the geom_violin() function in the ggplot R package (1). Small notches around the medians define the 95% confidence intervals for the medians, and dashed lines join means. The hydrophobic clustering of pre-LUCA Pfams is not significantly different from any of the other phylostrata, which may be due to the low number of per-LUCA Pfams. We note that unlike the animal proteins analyzed in James et al. (2), even the youngest, modern Pfams have clustering significantly below the expected value of 1 for independent sites (Binomial test; $p < 10^{-16}$).

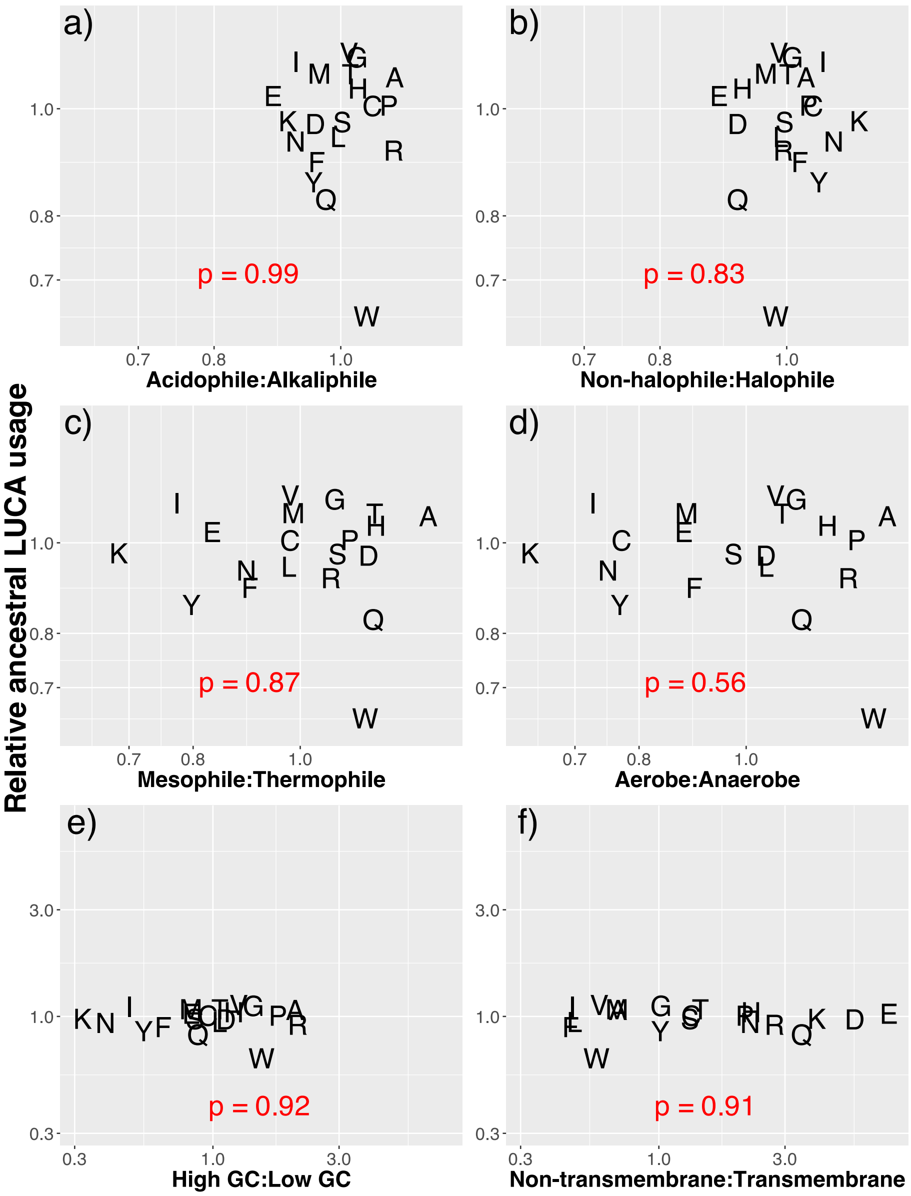

Figure S2. Environmental, genomic and structural amino acid biases do not correlate with the ancestral amino acid usage in LUCA clans. We compare relative amino acid usage in different conditions (x-axes) to that of our inferred ancient amino acid usage in LUCA clans (y-axis). Both axes are plotted on a natural logarithmic scale with back transformed tick labels. Different scales on the bottom row help show how LUCA enrichments are of larger magnitude than some factors (a-b), similar to some (c-d), and smaller than others (e-f). a-c) We calculated the proteomic amino acid usage as a function of environmental factors, using the proteomic and environmental data published by Amangeldina et al.(3). d) The oxygen tolerance of the prokaryotic species in the Amangeldina et al. (3) dataset was taken from the BacDive database (4). e) We compared the amino acid usage of organisms with > 60% GC to those with < 40% GC using GC content data from 11,710 representative prokaryotic genomes from the NCBI Refseq Prokaryotic Genomes database, taken from https://chrisgaby.github.io/post/prokaryotic-genome-size/index.html) (5) (accessed August 8th , 2024) . f) 1179 transmembrane Pfams (out of 8282 total Pfams) were identified based on the predicted presence of transmembrane alpha helices or beta sheets using DeepTMHMM (6). We compared the amino acid frequencies of non-transmembrane Pfams to those in transmembrane regions within transmembrane Pfams. Associated data can be found in 'Environment_AAC.csv', 'Bacdive_Oxygen_requirement.csv', 'prokaryotes_GC.csv' and 'DEEPTmhmm_consensusPfam.out' files on GitHub.

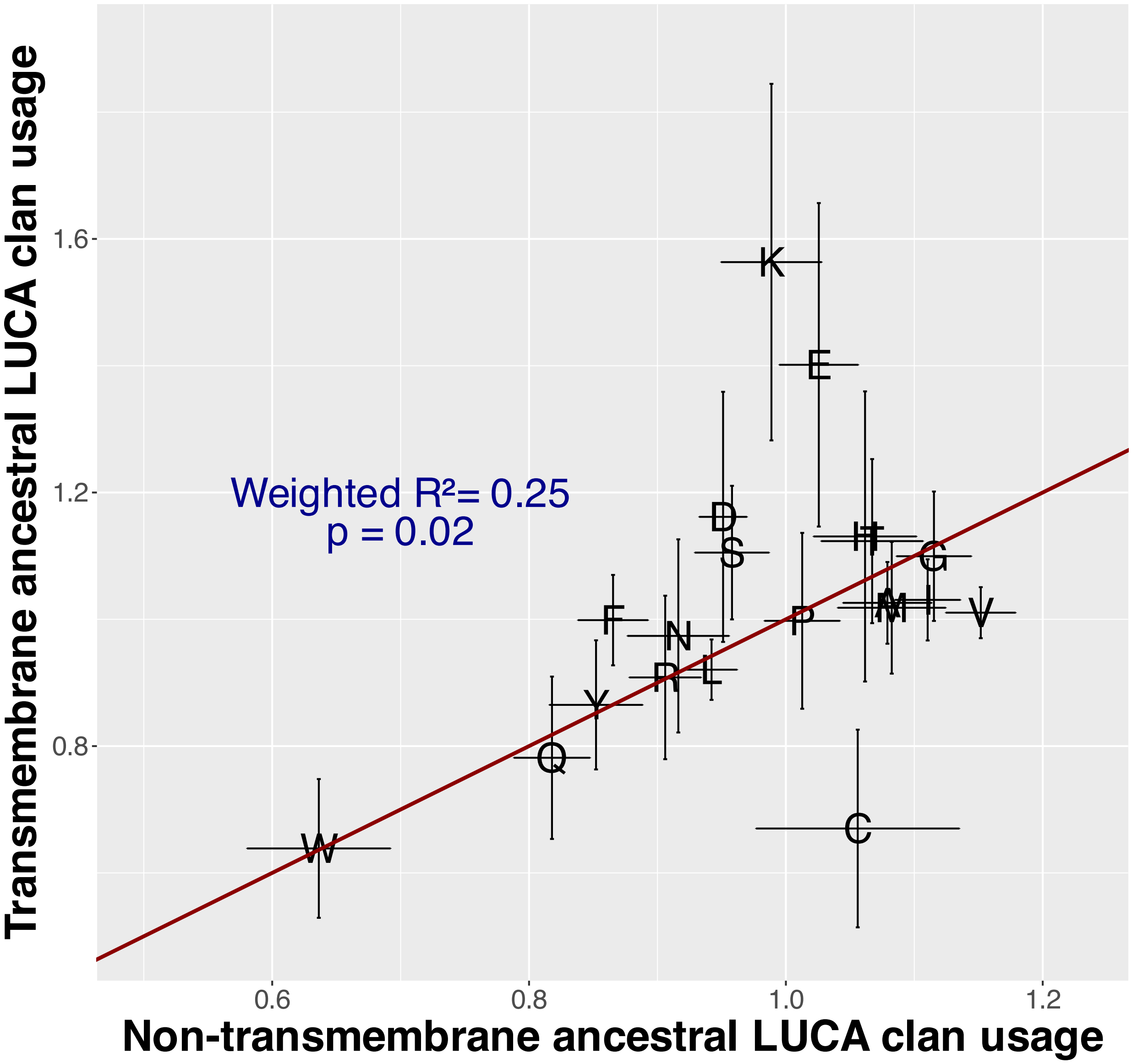

Figure S3. Ancient amino acid usage at transmembrane sites resembles that of non-transmembrane clans. LUCA and post-LUCA clans were used only if they contain either exclusively transmembrane or exclusively non-transmembrane Pfams. We calculated transmembrane amino acid frequencies only at transmembrane sites as predicted by DeepTMHMM (6). Error bars represent the standard errors of each LUCA / post-LUCA ratio, approximated via a Taylor expansion (7); see Methods. The R^2 and p value are estimated from a weighted model 1 regression, using the larger errors for transmembrane usage and neglecting errors in non-transmembrane usage. The y=x line is shown in red.

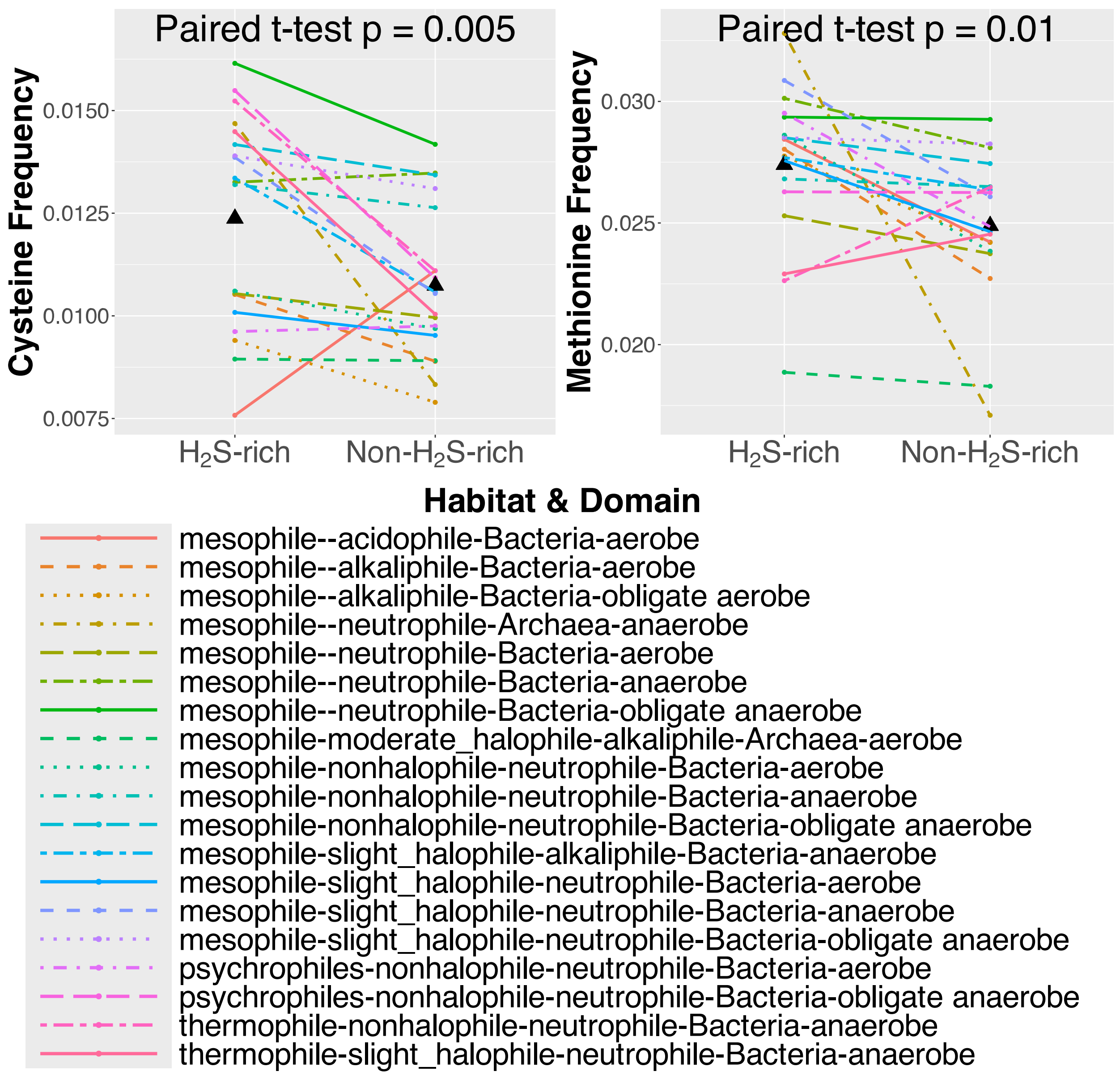

Figure S4. Sulfur-containing amino acids, M and C, are used more by contemporary prokaryotes living in H_2S-rich environments. Nineteen colored points and connected lines indicate matched pairs representing a range of environments (temperature, salinity, pH, and oxygen) and both Bacteria and Archaea. Triangles indicate means. We took data on habitat, taxonomy and amino acid frequencies from Amangeldina et al. (3). Each data point averages across a set of genera that inhabit similar environments and belong to the same taxonomic domain. One species was randomly sampled per genus to avoid pseudo-replication. Each line then joins the averages between the two sets of genera that inhabit otherwise similar environments and belong to the same taxonomic domain. Organisms living in H_2S-rich environments include sulfur-reducing and sulfur-oxidizing prokaryotes. A list of the species used in this analysis, with their respective environmental characteristics, and M and C frequencies can be found in 'sulfur_nonsulfur_species.csv' on GitHub.

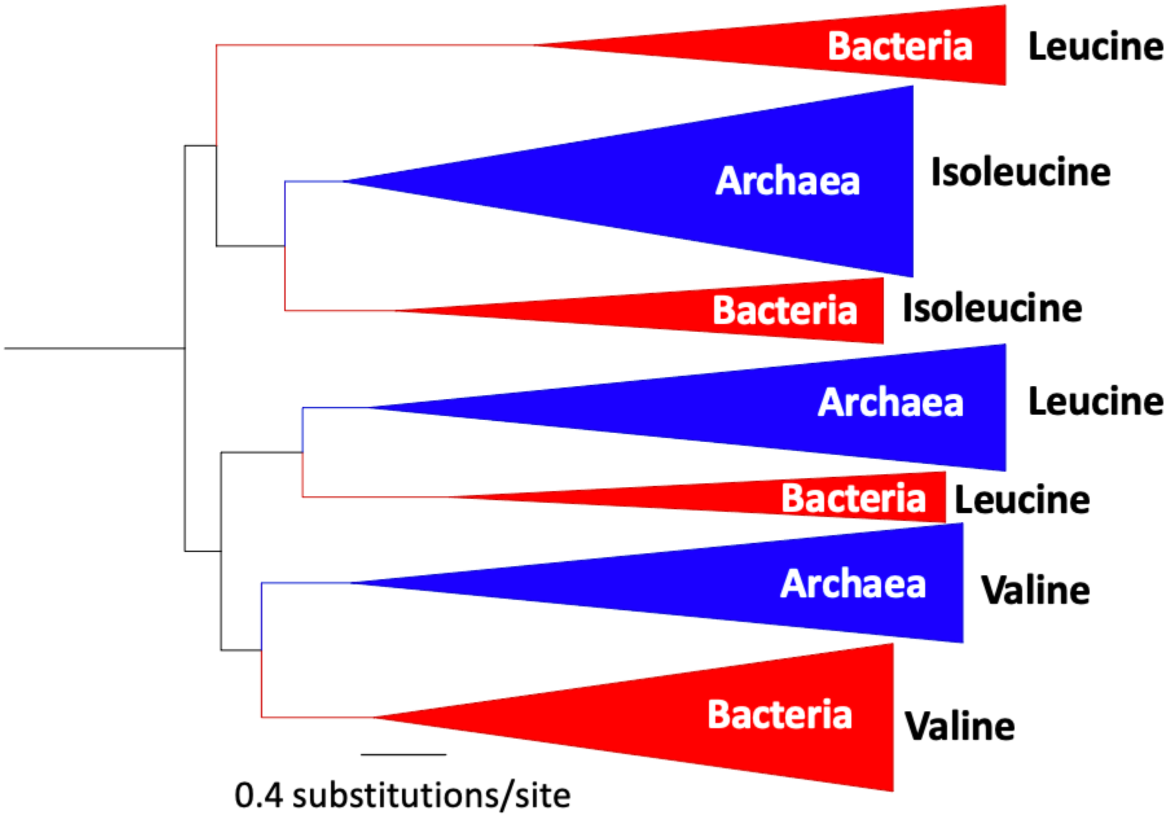

Figure S5. Topology of the leucine, valine, and isoleucine tRNA synthetase core catalytic domain confirms pre-LUCA origin. Three archaeal-bacterial splits denoting LUCA are shown, representing leucine, valine and isoleucine specific tRNA synthetase homologs. Collapsed archaeal clades names are in blue and collapsed bacterial clades are in red. Branch lengths were estimated using the Pfam-trained time non-reversible amino acid substitution model NQ.pfam (8), with an R10 heterogeneity model. Tree depicted is HGT-filtered, and midpoint rooted following pruning of the first ten branch length outliers.

Table S1. Our Pfam classifications confirm previous inference of LUCA's metabolism. All but two Pfams associated with the enzymes in the hydrogen (H_2) metabolism, assimilatory nitrate reduction, assimilatory sulfate reduction and Wood-Ljungdahl pathway were present in LUCA. Pfams associated with the nitrogenase family were not present in LUCA.

PATHWAY	KEGG ID	GENE NAME	PFAM IN LUCA	PFAM NOT IN LUCA
**Dihydrogen Metabolism**	K06281	hydrogenase large subunit	PF00374	
	K14126	F420-non-reducing hydrogenase large subunit	PF00375	
**Assimilatory Nitrate Reduction**	K00367	ferredoxin-nitrate reductase	PF04879;PF00384 PF01568	
**Assimilatory Sulfate Reduction**	K00957	sulfate adenylyltransferase	PF01507	
	K00392	sulfite reductase	PF01077;PF03460	
	K00930	phosphoadenosine phosphosulfate reductase	PF00696	
**Wood-Ljungdhal Pathway**	K00198	carbon-monoxide dehydrogenase catalytic subunit	PF03063	
	K05299	formate dehydrogenase (NADP+) alpha subunit	PF13510;PF12838 PF04879;PF00384	
	K15022	formate dehydrogenase (NADP+) beta subunit	PF14691;PF12838 PF04060;PF07992	PF14691 (LBCA)
	K22015	formate dehydrogenase (hydrogenase)	PF04879;PF00384 PF01568	
	K25124	FeS-containing electron transfer protein	PF13247;PF12800	
	K01491	methylenetetrahydrofolate dehydrogenase (NADP+)	PF02882	PF00763 (unclassifiable)
	K01500	methenyltetrahydrofolate cyclohydrolase	PF04961	
	K00297	methylenetetrahydrofolate reductase (NADH)	PF02219	
	K25007	methylenetetrahydrofolate reductase (NADH) small subunit	PF12225	
	K15023	5-methyltetrahydrofolate corrinoid/iron sulfur protein methyltransferase	PF00809	
	K00197	acetyl-CoA decarbonylase/synthase, CODH/ACS complex subunit gamma	PF03599;PF04060	
	K00194	acetyl-CoA decarbonylase/synthase, CODH/ACS complex subunit delta	PF03599	
**Nitrogen Fixation**	K02586	nitrogenase molybdenum-iron protein alpha chain		PF00148 (LBCA)
	K02587	nitrogenase molybdenum-cofactor synthesis protein NifE		PF00148 (LBCA)
	K02592	nitrogenase molybdenum-iron protein NifN		PF00148 (LBCA)
	K02588	nitrogenase iron protein NifH		PF00142 (unclassifiable)
	K02591	nitrogenase molybdenum-iron protein beta chain		PF00148 (LBCA);PF11844 (Proteobacteria)
	K02593	nitrogen fixation protein NifT		PF06988 (Proteobacteria)

Dataset 1 (separate file). Pfams with classified ages, ancestral amino acid frequencies, conserved sequence length, hydrophobic clustering score and clan data are also found in 'Pfam_data_ancestralAAC.csv' at sawsanwehbi/Pfam-age-classification GitHub repository.

Dataset 2 (separate file). Clans with classified ages, ancestral amino acid frequencies and maximum conserved sequence length are also found in 'Clan_data_ancestralAAC.csv' at sawsanwehbi/Pfam-age-classification GitHub repository

SI References

1. H. Wickham, *ggplot2: Elegant Graphics for Data Analysis* (Springer-Verlag New York, 2016).
2. J. E. James *et al.*, Universal and taxon-specific trends in protein sequences as a function of age. *Elife* **10** (2021).
3. A. Amangeldina, Z. W. Tan, I. N. Berezovsky, Living in trinity of extremes: Genomic and proteomic signatures of halophilic, thermophilic, and pH adaptation. *Curr Res Struct Biol* **7**, 100129 (2024).
4. L. C. Reimer *et al.*, BacDive in 2022: the knowledge base for standardized bacterial and archaeal data. *Nucleic Acids Research* **50**, D741-D746 (2021).
5. J. C. Gaby, Prokaryotic Genome Size. (2020).
6. J. Hallgren *et al.*, DeepTMHMM predicts alpha and beta transmembrane proteins using deep neural networks. *bioRxiv* 10.1101/2022.04.08.487609, 2022.2004.2008.487609 (2022).
7. H. Seltman, Approximations for mean and variance of a ratio. https://www.stat.cmu.edu/~hseltman/files/ratio.pdf (2012).
8. C. C. Dang *et al.*, nQMaker: Estimating Time Nonreversible Amino Acid Substitution Models. *Syst Biol* **71**, 1110-1123 (2022).
